# The Real Danger for Milk Protein Allergy: Developing a Scale for Family Doctors on Introducing Diet Diversity to Infants with Cow’s Milk Protein Allergy

**DOI:** 10.3390/nu17233770

**Published:** 2025-11-30

**Authors:** Celina Silvia Stafie

**Affiliations:** Preventive Medicine and Interdisciplinarity Department, University of Medicine “Grigore T. Popa” Iași, 700115 Iași, Romania; celina.stafie@umfiasi.ro

**Keywords:** cow’s milk protein allergy, CMPA, breastfeeding, dietary diversity, food allergy

## Abstract

**Background**: Cow’s milk protein allergy (CMPA) is one of the most common food allergies in infants, with a reported prevalence between 0.54% and 4.9%. In Europe, 1% of children are affected by CMPA in the first 2 years of life. Overdiagnosis leads to unnecessary dietary restrictions, with additional costs, while neglecting the diagnosis of CMPA has long-term repercussions on growth and development or, in the case of an acute reaction, may involve a life-threatening risk. The aim of this study is to develop a scale for family doctors to assess their practical knowledge of allergic pathology in newborns. **Materials and Methods**: To develop the scale, seven family doctors, each with over 10 years of practical experience, reached a consensus on the 20 items. The items were based on the guidelines of the European Academy of Allergology and Clinical Immunology on the prevention of food allergies in infants and young children. To verify the validity and internal structure of the scale, we conducted a pilot study on family doctors (N = 45), with a predominantly pediatric population, from one of the four university centers in Romania (Iași region). The results showed a good internal structure of the items in our scale. Content validity showed an I-CVI = 0.79 and an S-CVI = 0.91, which means that the scale can be considered excellent in terms of content. **Discussion**: The results of our study suggest that the new scale designed to assess the practical knowledge of family physicians in the field of allergic pathology in newborns, particularly with regard to cow’s milk protein allergy (CMPA), has solid psychometric properties. The excellent content validity, indicated by an item content validity index (I-CVI) of 0.79 and a scale content validity index (S-CVI) of 0.91, reflects strong agreement among experts on the relevance and comprehensiveness of the items included. **Conclusions**: The designed scale demonstrates excellent content validity and good internal structure, indicating that it is a reliable tool for assessing the practical knowledge of family physicians in the field of allergic pathology in newborns. This scale can support the improvement of the identification and management of cow’s milk protein allergy in primary health care.

## 1. Introduction

Cow’s milk protein allergy (CMPA) is one of the most common food allergies in infants, with a reported prevalence of between 0.54% and 4.9%. The figures vary from region to region and depending on the sample studied (infants fed naturally with breast milk, or artificially with formula milk). In Europe, 1% of children are affected by CMPA in the first 2 years of life [1,2,3]. For China, Yang et al. reported that in 2019, the prevalence of CMPA was 2.69%, and infants with CMPA had a strong family history of food allergy and atopy [4].

Overdiagnosis leads to unnecessary dietary restrictions, with additional costs. Neglecting the diagnosis of CMPA has long-term repercussions on growth and development and, in the case of an acute reaction, can pose a life-threatening risk [5,6,7,8,9]. Today, we are witnessing a veritable epidemic of food allergies, the first of which to appear chronologically is CMPA, contributing to the first manifestations of the sequence of events that are part of the “allergic march” [10].

The presence of a family history of allergy (regardless of the type of allergy) increases the risk of the child developing an allergic condition later in life. In general, having one parent with an allergy, predisposes the child to a 20–40% risk of developing an allergy [11]. This risk nearly doubles if both parents have allergies. Even in infants born to parents without allergies, there is a 15% chance that they will develop an allergy. Given these data, preventive strategies for CMPA are a topic of interest in current research [1,2].

Adverse reactions after ingesting cow’s milk can occur at any age, from birth, and even in exclusively breastfed infants, but not all of these reactions are allergic in nature. According to the nomenclature of allergies proposed in Europe in 2011 and approved by the World Health Organization (WHO), adverse reactions to milk, also called hypersensitivity reactions to milk, are classified as non-allergic, non-immune hypersensitivity (traditionally called “cow’s milk intolerance”) and allergic hypersensitivity to milk (or “cow’s milk allergy”, abbreviated CMPA) [12,13]. CMPA requires the activation of a mechanism underlying the immune system, as defined: “allergy is a hypersensitivity reaction initiated by specific immunological mechanisms” [1,14,15].

Immunoglobulin E (IgE) is a class of antibodies involved in allergic reactions, playing a key role in mediating rapid-onset allergic responses in conditions such as CMPA, where it triggers symptoms by reacting to proteins in cow’s milk. IgE-mediated CMPA is characterized by immediate allergic symptoms resulting from the interaction of IgE antibodies specific to cow’s milk proteins with mast cells, leading to allergic inflammation [7,16]. CMPA can be IgE-mediated and manifests with symptoms suggestive of atopy, associated or not with atopic eczema (dermatitis), allergic rhinitis, and/or asthma [3,14]. In the case of an IgE-mediated mechanism, the diagnosis is much easier to suspect because the symptoms are intense, impressive, and appear immediately (within the first 20 min or within a maximum of 2 h) after contact with or ingestion of cow’s milk proteins. Thus, the causal link is easily established and, in addition, the diagnosis is supported by standard allergy tests. Interpretation problems arise in the case of non-IgE-mediated CMPA (probably cell-mediated), when mainly gastrointestinal symptoms with late onset are present, sometimes after 48 h or even several days after ingestion, the mechanism being delayed [14,15,17,18].

Amino acid-based formulas are particularly indicated for IgE-mediated CMPA, but also for carefully selected cases of CMPA. Myths that extensively hydrolyzed formulas are nutritionally inadequate, must be firmly refuted [19,20,21].

Manufacturers must specify the source of protein in the formula: whey, or casein. The size of the peptides is important, as larger peptides are associated with higher allergenicity. Therefore, it is recommended to use a hydrolysate, with the highest percentage of peptides under 1000 daltons. The higher the degree of hydrolysis, the more bitter the taste, which manufacturers are constantly trying to improve, and the higher the price, which creates problems, if the purchase of the formula is not subsidized and parents have to provide special nutrition for their infants themselves [20,22].

Extensively hydrolyzed and delactosed formulas are intended for CMPA with secondary lactase deficiency. The lack of lactose, in addition to the unpleasant taste of the hydrolyzed formula, makes the palatability of this type of formula even more difficult to accept. In general, the younger the infant, the more likely they are to accept a particular formula [17,20,21,22].

Medical language serves as the exclusive means of accurate communication between colleagues and patients, requiring medical knowledge and guidelines to remain consistent. This uniformity is achieved through the widespread adoption of standardized terms, which are universally recognized with the same meaning across all professional groups [13]. The aim of this study was to develop a valid tool capable of assessing the level of expertise of family pediatricians in terms of CMPA diagnosis and subsequent therapeutic approaches. In addition, the study sought to assess the consistency of medical knowledge regarding key concepts such as cow’s milk allergy and the introduction of solid foods (also known as dietary diversity).

## 2. Materials and Methods

This is a pilot study conducted in 2023, between March and October. We used standardized interviews as our method.

### 2.1. Participants

The participants in the pilot study were family doctors (N = 45, 60% women) 27 women and 18 men, aged between 29 and 62 (M = 42.46, SD = 7.59), 73% of whom worked in general pediatric practice in the urban area of Iași (one of Romania’s four regional university centers), responsible for newborns in 17 neonatology departments. We divided the category of seniority in medical practice into four categories: less than 5 years, between 5 and 10 years, between 10 and 15 years, and more than 15 years of practice. In order to interpret the level of knowledge more accurately, we also divided the doctors according to the percentage of children aged 0 to 1 year, practically newborns, under their supervision from birth, into less than 20% newborns, between 20% and 30% newborns, and more than 30% newborns. In Romania, payment for family medicine services is made per capita and per service, and the payment is significantly higher for newborns, so family doctors are encouraged to include a higher percentage of newborns in their patient list.

### 2.2. Instrument and Procedure

Participation in this study was voluntary. Informed consent was obtained at the beginning of the study, and all 45 physicians agreed to respond to the questionnaire with the intention of contributing to research in this field. The questionnaire was administered in traditional paper format, with responses written in pen or pencil.

The scale for family doctors and pediatricians regarding the introduction of dietary diversity in infants was designed based on the guidelines of the European Academy of Allergy and Clinical Immunology (EAACI), which recommended approaches to prevent the development of immediate/IgE-mediated food allergies in infants and young children [5,6,12,23]. Data analyses for this pilot study were performed using IBM SPSS Statistics 22. Data analysis used the unstandardized scores of each participant.

To obtain content validity, we contacted seven experts (five women and two men) aged between 47 and 58, each with over 10 years’ experience in pediatric family medicine. The scale comprises four dimensions, as follows: the breastfeeding period dimension (items 1, 2, and 5), the introduction of dietary diversity in infants dimension (items 6, 7, and 8), the CMPA dimension and its effective application in practice (items 3, 4, 9, 11, 12, 13, 14, and 19), and The extent of allergies in newborns, infants, and children aged 1 to 3 years (points 15, 16, 17, 20). Question 10 is demographic, and question 18 is considered important by our 7 experts for any subsequent results using this scale. Due to the lack of responses to question 14, we had to exclude it from the entire analysis. Each multiple-choice question has a specific answer according to EAACI [23]. The open-ended items (11, 12, 14, 15, and 19) also have specific answers (according to EAACI), and their purpose was to provide more information about the physician and their understanding of CMPA in infants [24,25]. The scale can be found below (Table 1).

To verify the internal structure of our scale, we use item-total correlation in scale analysis [26]. To verify content validity, the 7 experts independently concluded that all items analyzed are relevant for assessing practical knowledge of allergic pathology in newborns. Their agreement was translated into the Item-level Content Validity Index (I-CVI) and Scale-level Content Validity Index (S-CVI) [19].

## 3. Results

The distribution of the respondents, family doctors, interviewed according to their seniority and the percentage of newborns in their care, and under their supervision, was as follows: in the category of less than 5 years of seniority, there are 5 doctors, of whom 2 have <20% newborns, 2 have between 20% and 30% and only 1 has >30% newborns. In the 5–10 years of experience category, there were 12 doctors, of whom 8 had between 20 and 30% newborns, and only 1 doctor had over 30% newborns under supervision. The most representative group was that of doctors with 10–15 years of experience, numbering 20, of whom more than half, 11 doctors, had 20–30% newborns in their care, 4 doctors had more than 30% and 5 doctors had <20%. In the last category of seniority, those with >15 years of experience, there are 8 doctors, and all have over 30% newborns, which suggests that in pediatrics, experience takes precedence in the choice of family doctor.

We correlated every item with the total score for its specific dimension. The results showed positive and signified correlation between r = 0.304, *p* = 0.05 and r = 0.710, *p* = 0.001 an follow: Item 1 r = 0.710, *p* = 0.001, Item 2 r = 0.650, *p* = 0.001, Item 3 r = 0.448, *p* = 0.001, Item 4 r = 0.376, *p* = 0.05, Item 5 r = 0.439, *p* = 0.001, Item 6 r = 0.605, *p* = 0.001, Item 7 r = 0.628, *p* = 0.001, Item 8 r = 0.502, *p* = 0.001, Item 9 r = 0.411, *p* = 0.001, Item 11 r = 0.446, *p* = 0.001, Item 12 r = 0.176, Item 13 r = 0.375, *p* = 0.05, Item 15 r = 0.637, *p* = 0.001, Item 16 r = 0.495, *p* = 0.001, Item 17 r = 0.304, *p* = 0.05, Item 19 r = 0.448, *p* = 0.001 and Item 20 r = 0.637, *p* = 0.001. The results revealed a valid criterion for item retention in our dimension-based scale analyses. The results showed an I-CVI = 0.79 and S-CVI = 0.91, which can be considered evidence of good content validity [27].

## 4. Discussion

Adverse reactions after consuming cow’s milk can occur at any age, from birth, and even in exclusively breastfed infants, but not all of these reactions are allergic in nature. According to the nomenclature of allergies proposed in Europe in 2011 and approved by the World Health Organization (WHO), adverse reactions to milk, also called hypersensitivity reactions to milk, are classified as non-allergic, non-immune hypersensitivity (traditionally called “cow’s milk intolerance”) and allergic hypersensitivity to milk or CMPA [12,16]. However, the literature has presented limited direct evidence regarding the existence of specific scales or standardized questionnaires designed exclusively to assess the introduction of solid food diversification in newborns with CMPA [28]. Therefore, the validation of our instrument (scale) represents a significant contribution to existing knowledge.

The results of our study suggest that the new scale designed to assess the practical knowledge of family physicians regarding allergic pathology in newborns, particularly cow’s milk protein allergy (CMPA), has solid psychometric properties. The excellent content validity, indicated by an item content validity index (I-CVI) of 0.79 and a scale content validity index (S-CVI) of 0.91, reflects strong agreement among experts on the relevance and comprehensiveness of the items included. This is consistent with recommendations emphasizing the importance of expert consensus in the development of clinical assessment tools to ensure their applicability and accuracy in the real world [27,29].

The good internal structure demonstrated by the scale further supports its reliability as an assessment measure. Internal consistency is crucial to ensure that the items consistently assess the desired construct—knowledge of allergic pathology—thus making the scale suitable for identifying knowledge gaps among family physicians. Such identification is essential because CMPA is a common condition in infants, with an estimated prevalence of between 0.5% and 5%, and early detection is crucial to prevent both overdiagnosis and underdiagnosis, which can lead to unnecessary dietary restrictions or serious health complications, respectively [4,30].

Thus, basing the scale on the guidelines of the European Academy of Allergy and Clinical Immunology reinforces its clinical relevance and alignment with current standards in the prevention and management of allergies in infants. Given the challenges associated with diagnosing CMPA—where symptoms are often nonspecific and overlap with other conditions—the scale may enable primary care physicians to make more informed clinical decisions and timely referrals, which is consistent with the literature emphasizing the importance of education and training in primary care to improve allergy outcomes [4,31].

Furthermore, the implementation of our scale could mitigate the consequences associated with misdiagnosis. Overdiagnosis has been associated with unnecessary elimination diets, which pose nutritional risks and socioeconomic burdens, while underdiagnosis can lead to growth disorders or even life-threatening reactions in infants [8,9,31].

Thus, by improving the assessment of physicians’ knowledge specifically tailored to allergic pathologies in newborns, the scale promises to promote improved management strategies in primary care, which could lead to better growth, development, and quality of life for affected children [32].

Question 14-a concerns total IgE, which is important in clinical practice. Total IgE is important in family medicine because it represents a way of “assessing a child’s allergy”, a kind of “scale” for allergies. The higher the total IgE value at birth, the more certain the doctor will be that the child, based on genetic inheritance and certain environmental conditions, will have a high risk of developing CMPA or other forms of food allergies. The total IgE value, immunoglobulins which are actually antibodies, increases proportionally to the number, density per unit of time, but also to the severity of the child’s allergic reactions.

Question Q14-b, the second part of question Q14, was an open-ended question, which respondents could elaborate on depending on their level of knowledge about food allergies in newborns and young children. From a clinical point of view, the two types of allergies, IgE-mediated and non-IgE-mediated, can manifest themselves in a similar way: erythematous, pruritic, migratory rashes in IgE-mediated cases and rather fixed rashes in non-IgE-mediated cases. Why is it important for family doctors caring for allergic newborns to have this knowledge? Food allergies in young children and allergies to cow’s milk protein have an IgE-mediated mechanism with anaphylactic potential. Because they can be detected early by collecting total IgE from the umbilical cord at birth, and if the total IgE value in the umbilical cord is >5–10 IU/mL and if the child has an allergic parent, then the family doctor already knows that they have a newborn at risk.

For at-risk newborns, the family doctor has two measures to take: to recommend exclusive breastfeeding or hypoallergenic milk formulas, possibly partially hydrolyzed, and to recommend that the introduction of solid foods, or diversification, begin after 7–8 months, with foods being introduced every 7–10 days (except for exotic foods or those in the 6 allergenic categories), and not every 2–3 days.

A family doctor who understands the immunological mechanisms of CMPA, knows that a mother who feeds a newborn with cow’s milk allergy, either initiates early diversification with allergenic foods, or exposes the child to food allergies, respiratory allergies, or anaphylactic shock.

Future research should attempt to validate this scale in larger and more diverse populations of primary care providers, to confirm its generalizability and predictive value. In addition, examining how the use of the scale influences clinical practice and patient outcomes, would provide valuable information about its practical utility. Integrating such assessment tools into continuing medical education, could further raise the standard of care in the management of CMPA and associated allergic conditions in infancy.

If we analyze the practical consequences behind the answers to questions 15–17, we could say that family doctors are unsure, or have decided to impose a certain diet. The reasons may be difficulties in implementing a restrictive diet from the mother’s point of view (large family, lack of time or availability), or fear for the child’s immune tolerance. However, data from the literature provide a precise timeline and medical evidence for the exclusion diet [1,5,7].

The cow’s milk protein exclusion diet is prescribed for a maximum of two to four weeks. The physician must also be aware of the negative psychological effects of the elimination diet, in addition to the financial and palatability effects of special formulas.

Elimination of cow’s milk protein from the mother’s diet should be recommended as a first step in breastfed infants with suspected CMPA. However, the mother should be counseled nutritionally, with calcium and vitamin D supplementation and written specification of milk-containing foods [1,17].

For formula-fed infants, unless the clinical picture suggests a severe IgE-mediated allergy, the first dietary therapeutic option should be an extensively hydrolysed protein milk formula. In children who are also suspected to have a CMPA-related post-enteritis syndrome with digestive mucosal lesions and secondary lactase deficiency, extensively hydrolyzed and lactulase-deficient formulas are indicated. When the described manifestations suggest CMPA-related anaphylaxis, amino acid-based formulations are prescribed [17,22]. If the exclusion diet has been strictly adhered to and the symptoms do not improve, then it is not related to CMPA, and differential diagnostic problems arise. It may not be an allergy but, rather, another disease, or the child may have an allergic pathology; the culprit for the allergy may not be milk, but perhaps soy or egg, these two allergens being among the most common culprits in food allergy in children in Europe [12]. If symptoms improve after the elimination diet, the diagnosis of APMC is likely.

### Importance, Implications, and Benefits

The proposed scale can demonstrate its importance for researchers when they have to assess the actual level of knowledge on the three most important dimensions of care of the newborn at risk and the newborn with cow’s milk protein allergy: breastfeeding, introduction of diet diversity (optimal timing and content), and specific measures for CMPA.

Once adopted in practice, this scale can demonstrate that there are differences between the level of correct knowledge and the level of correct application in practice of family physicians, regarding the care of the newborn with CMPA, which may negatively affect the health status of the allergic infant, with long-term consequences.

Also, the medical errors committed between 0 and 1 year have direct and decisive consequences on the quality of life of these children and their families, with future impairment of allergic sensitization, as in the case of diversification of inadequately early solid food or allergenic food, in the case of babies with CMPA.

The implications and benefits of using this scale in medical practice are self-assessment of knowledge, through periodic application of the scale, for the purpose of continuing medical education. Refresher courses can then be followed to improve and update scientific knowledge according to current allergology guidelines, but also for the purpose of evaluating how this knowledge is applied in practice, personalized according to the history of allergic disease of each patient.

## 5. Conclusions

In conclusion, this scale constitutes a reliable and content-valid instrument to assess family doctors’ practical knowledge of CMPA and allergic pathology in newborns. Its adoption in primary care settings could contribute significantly to bridging the knowledge gap, facilitating early and accurate diagnosis, and promoting evidence-based management of cow milk protein allergy in infants.

## Figures and Tables

**Table 1:** Scale for family doctors and pediatricians on introducing diversification to babies with CMPA.

Questions and Answers
(1) What breastfeeding period do you recommend for mothers? Choose one of the options below: (a) I do not recommend a specific period (b) minimum 6 months (c) minimum 4 months (d) minimum 9 months
(2) Do you recommend the addition of powdered milk to supplement breast milk? (a) yes (b) no (c) cow’s milk (d) solid food
(3) How do you define an allergic child? You can choose 1 or more of the following options: (a) A child of allergic parents (b) A child of parents/grandparents with diabetes (c) A child who has frequent episodes of hives from food (d) Children with an allergy to cow’s milk proteins (CMPA) (e) Children with frequent colds during the cold season
(4) In the case of allergic children, what kind of milk powder do you use? (a) Humana milk powder (b) Hypoallergenic milk powder Alfare, Althera (from Nestle) (c) Alfamino (from Nestle) hypoallergenic powdered milk (d) List one or two brands of milk powder you have used for this purpose
(5) When do you recommend introducing solid foods? Choose one of the options below: (a) from 4 months to 6 months (b) from 6 to 7 months (c) after 7 months but before 12 months (d) after 12 months (e) whenever accepted by the infant
(6) Which foods do you start with? You can choose 1 or more of the following: (a) carrot, celery, parsnip (b) carrot, parsnip (c) grated apple (d) jars of ready-prepared food from baby food companies (e) whole egg/egg yolk/egg white (f) chicken meat/chicken filet (g) potato (h) cow’s cheese
(7) At what age do you start diet diversification for an allergic child? Choose one of the options below: (a) 4 months (b) 6 months (c) 9 months (d) 12 months
(8) Are there any restrictions regarding dietary diversification for allergic children? (a) From (b) No
(9) Which foods are most likely to cause allergic reactions in infants 0–1 years? You can choose 1 or more of the following options: (a) Milk (b) Eggs (c) Meat (d) Peanuts (e) Fish (f) crustaceans
(10) How many cases of CMPA have you had so far?
(11) How do you diagnose CMPA?
(12) What kind of diet do you recommend for children with CMPA?
(13) Which foods are not recommended for allergic children under the age of 1? You can choose 1 or more of the following options: (a) Fish (b) Egg (c) chocolate (d) soya (e) nuts (f) hazelnuts (g) cashew nuts (h) meat
(14-a) What is the significance of the total IgE value? (14-b) In your opinion, what are the practical differences between IgE-mediated allergy and non-IgE-mediated allergy?
(15) What is an allergenic avoidance diet?
(16) Is the allergen avoidance diet useful in preventing allergic diseases? (a) Yes (b) No
(17) If you use an allergenic avoidance diet, how long do you maintain it? (a) 2 months (b) 6 months (c) 1 year (d) for life
(18) Do you have cases of allergic children for whom allergen avoidance could not be applied? (a) Yes (b) No
(19) What is the clinical consequence in children who cannot avoid the allergens that are incriminated in allergic symptoms (urticaria, rhinitis, asthma, atopic dermatitis)?
(20) The only safe and effective method to find out which allergens give symptoms of allergic disease is skin testing or bronchial challenge. Do you consider these two exploratory methods useful? (a) Yes (b) No (c) I do not recommend them (reasons)

## Data Availability

The original contributions presented in this study are included in the article. Further inquiries can be directed to the corresponding author.
